# Cross-species mapping of bidirectional promoters enables prediction of unannotated 5' UTRs and identification of species-specific transcripts

**DOI:** 10.1186/1471-2164-10-189

**Published:** 2009-04-24

**Authors:** Helen Piontkivska, Mary Q Yang, Denis M Larkin, Harris A Lewin, James Reecy, Laura Elnitski

**Affiliations:** 1 National Human Genome Research Institute, National Institutes of Health, Rockville, MD, 20852, USA; 2 Department of Biological Sciences, Kent State University, Kent, Ohio 44242, USA; 3 Department of Animal Sciences, University of Illinois at Urbana-Champaign, Urbana, IL 61821 USA; 4 Institute for Genome Biology, University of Illinois at Urbana-Champaign, Urbana, IL 61821, USA; 5 Department of Animal Science, Iowa State University, Ames, IA 50011, USA

## Abstract

**Background:**

Bidirectional promoters are shared regulatory regions that influence the expression of two oppositely oriented genes. This type of regulatory architecture is found more frequently than expected by chance in the human genome, yet many specifics underlying the regulatory design are unknown. Given that the function of most orthologous genes is similar across species, we hypothesized that the architecture and regulation of bidirectional promoters might also be similar across species, representing a core regulatory structure and enabling annotation of these regions in additional mammalian genomes.

**Results:**

By mapping the intergenic distances of genes in human, chimpanzee, bovine, murine, and rat, we show an enrichment for pairs of genes equal to or less than 1,000 bp between their adjacent 5' ends ("head-to-head") compared to pairs of genes that fall in the same orientation ("head-to-tail") or whose 3' ends are side-by-side ("tail-to-tail"). A representative set of 1,369 human bidirectional promoters was mapped to orthologous sequences in other mammals. We confirmed predictions for 5' UTRs in nine of ten manual picks in bovine based on comparison to the orthologous human promoter set and in six of seven predictions in human based on comparison to the bovine dataset. The two predictions that did not have orthology as bidirectional promoters in the other species resulted from unique events that initiated transcription in the opposite direction in only those species. We found evidence supporting the independent emergence of bidirectional promoters from the family of five RecQ helicase genes, which gained their bidirectional promoters and partner genes independently rather than through a duplication process. Furthermore, by expanding our comparisons from pairwise to multispecies analyses we developed a map representing a core set of bidirectional promoters in mammals.

**Conclusion:**

We show that the orthologous positions of bidirectional promoters provide a reliable guide to directly annotate over one thousand regulatory regions in sequences of mammalian genomes, while also serving as a useful tool to predict 5' UTR positions and identify genes that are novel to a single species.

## Background

The completed sequence of numerous vertebrate genomes has enabled rapid gene annotation across species using orthologous relationships. This approach is feasible because purifying selection, acting on the open reading frames of coding exons and aimed at preserving encoded protein sequences, minimizes the sequence divergence that can occur. The sequences of these protein-coding genes generally change more slowly over millions of years than do non-coding sequences. Similarity at the nucleotide level is reflected in the likeness of structure and function of the gene products produced in different species. Additional features, such as non-coding functional elements, are also maintained as conserved sequences across species through the action of purifying selection [1]. Enhancer elements are often predicted from their distinctive sequence conservation. Other functional classes, such as promoters, contain more plasticity in their composition and do not lend themselves to identification in this manner. Given that precise computational methods are not yet developed for predicting promoter regions in newly assembled genomes, their annotation lags behind that of coding genes and enhancers.

We hypothesized that promoter regions could be reliably mapped across species using a unique class of promoter that is flanked by genes on each side. These promoters, known as bidirectional promoters, would be useful for annotating promoter regions across mammals because the genes on both the left and right sides of the promoter change slowly. Thus, the promoter region is maintained as a recognizable, intergenic, architectural region that is amenable to computational discovery. Furthermore, if no repetitive elements were inserted at the bidirectional promoter region in either species, the intergenic distances should be maintained across species. To lend support to this hypothesis, Takai and Jones (2004) [2] showed the exclusion of repetitive elements from bidirectional promoters of human chromosomes 20, 21, and 22.

Bidirectional promoters were originally defined as the regulatory regions present in the intergenic space of two oppositely oriented genes whose transcription start sites (TSSs) were separated by no more than 1,000 bp [3]. Such genes appear in a head-to-head arrangement, i.e. facing away from one another, and are transcribed from opposite strands of DNA. The closely spaced arrangement of the TSSs flanking the bidirectional promoter was recognized as a non-random event, proven by the fact that a greater-than-expected number of genes had this architecture [2]. Up to 10% of human protein-coding genes were initially identified with bidirectional promoters. We subsequently identified thousands of additional, putative, bidirectional promoters by analyzing divergently transcribed, spliced EST data [4]. The methodology of mapping bidirectional promoters across species used here treats the genes on each side of a promoter as anchors that delimit the intergenic, orthologous regulatory region. If the genome of the other species contains conserved gene order and orientation at the orthologous location, then the intergenic promoter region must have evolved from the ancestral sequence at that location. If the intergenic distance of the annotated transcripts in the other species is also maintained as ≤ 1,000 bp, the orthologous bidirectional promoter is declared validated. Of added benefit, this method is not dependent on the level of nucleotide sequence conservation in the promoter regions, which can vary extensively [5].

The enrichment of bidirectional promoters in the human genome evokes questions about their evolution. In some cases, chromosomal rearrangements could have conjoined promoter regions of two genes. Those genes would remain united through all subsequent speciation events due to selective pressure against change. Any breakage of the union (within or near the bidirectional promoter) could disrupt the normal regulation of both genes, potentially having profound (disadvantageous) effects on cellular function. If true, bidirectional promoters should provide an evolutionary timestamp of rearrangement events across mammalian genomes. Alternatively, some unidirectional promoters could have lost control of their regulated transcription, enabling RNA polymerase to load and traverse in the opposite direction [6]. This scenario could serve as a mechanism for generating *new *genes in the genome, which would occur in a rare and species-specific manner.

Building on our previous computational infrastructure, we utilize updated human genome annotations to compare bidirectional promoters in human and bovine genomes to test the hypothesis that long-term evolutionary histories of these promoters could be identified and used to annotate the bovine genome. We used these data to create a detailed regulatory map of orthologous promoter regions across 5 placental mammals (human, chimp, cow, mouse and rat). As an outcome of the analysis, we have shown that the "locked" arrangement of genes around these promoters enables prediction of unannotated 5' UTRs using cross-species comparisons. Furthermore, we identified bidirectional promoters that lack orthologous counterparts in all other species, supporting the conclusion that species-specific genes can be identified from rigorous, cross-species comparisons of this dataset. One human-specific example was from the family of five RecQ helicase paralogs (*WRN*, *BLM*, *RECQL*, *RECQL4*, and *RECQL5*), all of which have bidirectional promoters that developed independently.

## Results

### Mapping bidirectional promoters in the cow genome

Bidirectional promoters were predicted in the cow genome from *in silico *analyses of gene order, orientation and intergenic distances, analogous to our studies in the human and mouse genomes [4,7]. The official bovine gene set (OGS v1, available at the Bovine Genome Database; [8]) contained 4,948 bidirectional gene pairs. Those predictions were made without the normal requirement for an intergenic distance of ≤ 1,000 bp due to incomplete annotation of 5' UTRs in the cow genome. Thus, we labeled the data as "low-stringency" predictions. In comparison, our previous studies in the human and mouse genomes identified 5,000–6,000 bidirectional promoters whose intergenic distances were limited to 1,000 bp (i.e., "high stringency") [4,9], suggesting that the number of bidirectional promoters possible in the cow genome was as high as 6,000. We concluded that our low stringency bovine set captured a majority of actual bidirectional promoters, but that limited annotations contributed false positive and false negative predictions.

To further assess the bidirectional promoters in the cow genome, we examined additional transcript evidence. The OGS v1 dataset, which lacked information regarding most 5' UTRs of bovine genes, was supplemented with RefSeq annotations from GenBank and spliced EST data from the UCSC Cow Genome Browser. Together these datasets identified 1,574 bovine bidirectional promoters, all of which met the requirement of no more than 1,000 bp separating any pair of TSSs (Supplemental Figure S13 in [10]).

**Figure 1:**
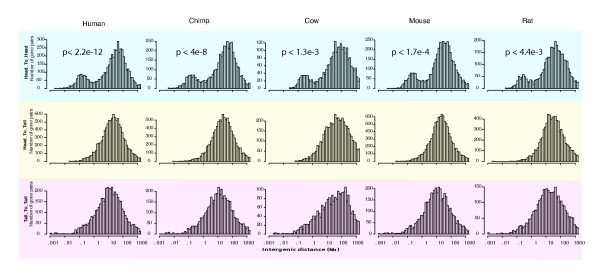
**Intergenic distances in human, chimp, cow, mouse, and rat genomes**. Intergenic distances were recorded from RefSeq annotations in each genome. The data are plotted as a comparison of head-to-head, head-to-tail or tail-to-tail gene arrangements. Intergenic distances of 1,000 bp or less in the head-to-head gene arrangement represent bidirectional promoters. P-values shown in each plot denote the significance of the number of genes found in the head-to-head group compared to the other categories at a distance of 1,000 bp or less, using a Chi-square test of independence.

We hypothesized that bidirectional promoters form a core regulatory structure in all mammalian genomes. To assess this hypothesis quantitatively, the intergenic distances for all promoters having a "head-to-head" configuration were examined in the human, chimp, cow, mouse, and rat genomes (Fig. 1). For comparison, we examined "head-to-tail" genes that are arranged in a parallel orientation and "tail-to-tail" genes whose 3' ends are adjacent. These data showed a significant enrichment for the head-to-head arrangement within 1,000 bp compared to the same distance in the other datasets (p-value < 2.2e-12 in the human dataset and p < 1.3e-3 in all datasets, respectively, for the χ^2 ^test).

### Human bidirectional promoters have orthologs in cow

To address a core set of orthologous bidirectional promoters in mammals, we mapped positions of the regulatory regions in the human and cow genomes. We noted that less than 25% (1,369) of the human bidirectional promoters controlled expression of genes that encode proteins. The remainder regulated combinations of protein-coding genes with RNA gene partners or pairs of RNA genes. We proceeded by mapping only these 1,369 human promoters from the protein coding set, because of the limited annotations of RNA genes available in most mammalian species.

**Figure 2:**
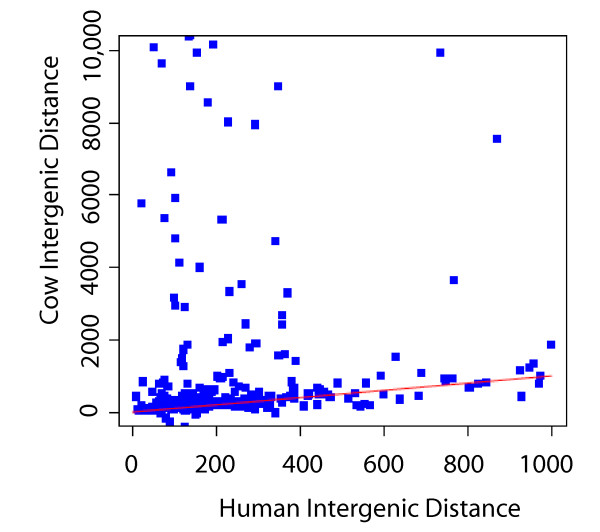
**Intergenic distances between gene-pairs mapping to orthologous positions in human and cow**. Promoters in the human dataset were limited to an intergenic distance of < 1,000 bp, whereas the intergenic distances at the orthologous bovine promoters were unlimited. Orthologous gene pairs with the same relative distance in each species fall alongside the red diagonal line (i.e., where X = Y).

When compared between human and cow, 94% of the regions mapped to an orthologous position at one or both of the genes flanking each promoter. Moreover, the intergenic distances of orthologous bidirectional promoters were found to be similar for the majority of gene pairs (Fig. 2). Nevertheless, despite the presence of orthology, some intergenic distances in cow were much larger than the allowable 1,000 bp, providing an opportunity to assess if the differences represented biological diversity or technical anomalies.

**Figure 3:**
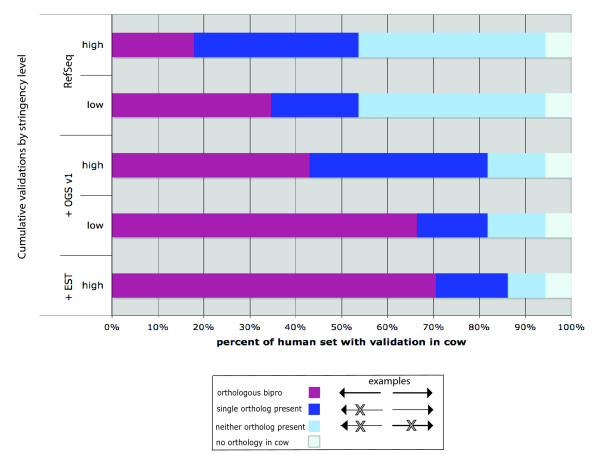
**Cumulative validation of bidirectional promoters in the cow genome**. A set of confirmed bidirectional promoters from the human RefSeq annotations (Hg18 assembly) was mapped to orthologous positions in the cow genome. If the cow transcript data independently identified a bidirectional promoter at that same location (with ≤ 1,000 bp intergenic distance) these predictions were considered validated (purple bars). Otherwise the predictions were classified as low stringency (i.e., > 1,000 bp). The validation data are additive, accumulating information from the top to bottom of the image (i.e., from RefSeq, high stringency data to EST, high stringency data, respectively). Dark blue and and medium blue bars designate promoters that lack annotation for one or both of the two genes in the pair, respectively. Light blue bars designate gene pairs lacking an ortholog of either gene in the pair.

Individual gene pairs were examined for transcript evidence at the orthologous locations in cow. Those transcripts were required to uphold the 1,000 bp intergenic distance rule to validate the location as an orthologous bidirectional promoter. RefSeq annotations in cow provided validation for 20% of the 1,369 human promoters (Fig. 3). An additional 7% of the cross-species predictions were validated from the OGS v1 annotations. Supplementing this comparison, the spliced EST annotations from cow confirmed another 5% of the orthologous promoter predictions. In total, 479 human promoters were validated in cow through a combined assessment of orthology and transcript annotations. An additional 487 human bidirectional promoters were supported at orthologous locations by the low-stringency predictions in the cow genome. These data confirmed that conserved synteny was maintained at regions orthologous to human bidirectional promoters; however, validation of those regions as true bidirectional promoters was hindered by the possibility of missing data at the 5' ends of genes in cow. Of the remaining comparisons, the majority were present at orthologous locations, but lacked an annotation for one of the two genes in the pair (200 regions; Fig. 3, dark blue bars) or for both genes (121; medium blue bars). Only 6% of the gene pairs had no record of orthology in the cow genome (82; light blue bars).

### Cross-species UTR prediction

Missing UTR information in the bovine genome could be addressed using complementary evidence from multiple gene annotations. Over half of the bidirectional promoters predicted using cow OGS v1 annotations were the low stringency type, with an intergenic distance greater than 1,000 bp. Comparison to the high-stringency EST data identified 5'UTRs for the genes in this data that brought neighboring TSSs closer together. These UTRs were located at genomic distances of up to 100,000 bp upstream of the characterized OGS v1 coding exons. In a pilot experiment, we addressed the large intergenic distances found in cow relative to human by examining ten gene-pairs that had extremely large intergenic distances in cow. The genes from each pair were spaced greater than 50 kb apart at their 5' ends, compared to a 1 kb intergenic distance in human (as seen in Fig. 2, see also Table 1). All ten of the gene pairs were re-annotated to update their 5' UTRs (Otterlace annotation pipeline, [11]) and nine of ten were subsequently validated as bidirectional promoters in the cow genome. Following the addition of the UTR annotations, a comparison of the cow and human genes showed uniformly long first introns, averaging 88,844 bp. These long distances contrasted sharply with the short distances being maintained between the TSSs of each set of genes. Thus, manual curation performed for 10 cow genes confirmed that unannotated 5' UTRs accounted for the failure of 9 of those regions to be validated as orthologous promoters in cow in our original comparison. Similar evidence for missing data at the 5' UTRs was found in an analysis of the full dataset (Additional file 1). For example, human promoters were mapped to the cow genome and validated using cumulative evidence from each of the cow gene sets. Given that the reference gene annotations are maintained as separate resources, we found they contain complementary as well as unique information that supported our claims. The OGS v1 data, which has a minimal number of 5' UTR annotations, gained UTRs from RefSeq or EST data. Despite our best efforts, a large number (604) of OGS v1 gene predictions failed to be validated in bidirectional promoters because the EST or RefSeq datasets lacked entries for those genes, confirming that the bovine transcriptome data is incomplete.

**Table 1:** Updates to the cow bidirectional promoter annotations predicted from cross-species comparisons

Chrom	Start	Stop	Gene	Extension length (genomic bp)	Species updated
2	20224157	20407027	*NFE2L2*	151,340	cow

4	16111942	16252609	*ASNS*	131,007	cow

12	26013752	26135022	*HMGB1*	120,256	cow

11	57090805	57198155	*LOC51057*	94,165	cow

2	34331018	34438555	*LOC130940*	85,750	cow

23	5187362	5271454	*G3BP2*	81,425	cow

15	70375828	70441308	*CUGBP1*	60,311	cow

7	84083382	84169517	*MGC34713*	58,429	cow

10	49158540	49417244	*TEX9*	54,675	cow

1	92194924	92250343	*SERPINI1*	51,488	cow

A reciprocal examination of orthology at bidirectional promoters mapped cow bidirectional promoters to the human genome. Ninety percent of bidirectional promoters in cow mapped to the human genome at orthologous locations. A small number contained an intergenic distance that was too large to be called a bidirectional promoter in the human gene pair. Six of these regions were chosen for manual curation. Five of the six were confirmed as bidirectional promoters upon re-evaluation of the available transcript information (using the Otterlace annotation pipeline [11]) (Table 2). The new annotations were recorded in the 2008 Vega human gene annotations [12].

**Table 2:** Updates to the human bidirectional promoter annotations predicted from cross-species comparisons

Chrom	Start	Stop	Gene	Extension length (genomic bp)	Species updated
7	7188878	7250231	*C1GALT1*	193,392	Human

17	37247576	37258122	*KLH10*	2,113	Human

19	50680391	50691931	*FLJ40125*	1217	Human
4	57539791	57592081	*POLR2B*	1,098	Human

19	54095428	54118339	*NUCB1*	309	Human

12	54885526	54901880	*OBFC2B*	120	Human

### A unique bidirectional promoter in cow

Genomic rearrangements that displaced one gene from an orthologous bidirectional gene pair occurred in less than 1% of the genes analyzed. An example of such an event was found when the bidirectional promoter for cow *CYB5R4 *(cytochrome b5 reductase 4, this represents an alternative promoter of *CYB5R4*. Through alternative splicing, the first exon is also used for a different gene, *RIPPLEY2*). did not validate in human. The human region contained the ortholog for *CYB5R4*, but not the partner gene from cow (GenBank Accession DV834581). This partner was expressed in numerous cow expression libraries from the brain (Bovine Genome Sequencing Program: Full-length cDNA Sequencing, unpublished) and although it had a minimal open reading frame, it showed strong evidence for RNA secondary structure (Supplemental Fig. S15 in [10]). The unique appearance of the transcript in cow was explained by a 43 Mb chromosomal inversion on cow chromosome 9 ([10], Supplemental Fig. S14 in [10]). The transcript DV834581 crossed this rearrangement breakpoint. None of the other sequenced mammalian genomes showed evidence of the 43 Mb inversion. A duplicated MIR3 SINE was found flanking the inversion on both sides, with one copy being embedded within exon 3 of the DV834581 transcript. Although the repetitive element was implicated in the inversion, no clear model explained its role mechanistically. Conserved synteny between the human, macaque, chimp, mouse, rat, dog, and pig genomes indicated that no other genomes could reconstitute this transcript because it spanned the unique chromosomal junction. Therefore, the transcript DV834581 was identified as a bovine-specific transcript being transcribed from a bidirectional promoter that was not bidirectional in any other species.

### Parallel evolution of bidirectional promoters regulating RecQ helicases

**Figure 4:**
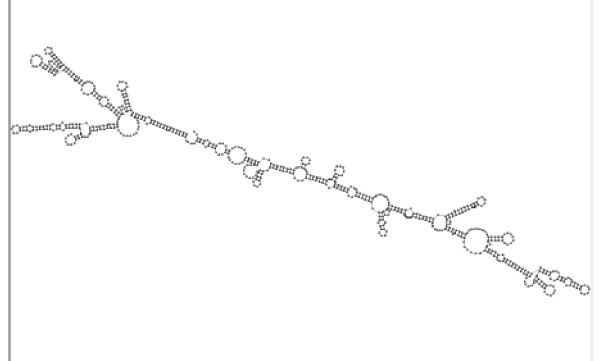
**Folded structure of the *BLM *partner gene**. Most expressed transcripts from this locus lack a significant open reading frame indicating that it could function as an RNA gene. Strong, orderly, secondary structure is detected in the processed transcript.

The presence of the unique bidirectional promoter in cow suggested that novel transcripts could be identified in any mammalian genome using our comparative approach. We therefore examined the human genome for evidence of novel RNA transcripts regulated by bidirectional promoters that were not predicted in other species. We found that the bidirectional promoter of the Bloom Syndrome gene (*BLM*) regulated a candidate human-specific transcript. With limited coding potential, reliable expression evidence, and strong secondary structure (Fig. 4) this transcript (e.g., GenBank Accession BG472948) was generated from a chromosomal region that aligns at the nucleotide level in several mammals, from human to opossum, suggesting an evolutionary history of at least 170 million years [13]. However, evidence for transcription from this site was limited to human. Exons of this transcript exhibited sequence identity between human and chimp, but not in more distant primate genomes. Furthermore, no compensatory mutations were present in the exonic regions of more distant primate sequences, which would have indicated that an RNA gene was functional and under selection in those species. A variant containing a small open reading frame under purifying selection in human and chimp (as evidenced by the excess of synonymous nucleotide changes compared to nonsynonymous changes, *d*_*S *_> *d*_*N*_) did not align to the marmoset genome (i.e., Catarrhini-specific). We concluded that any coding potential of the transcript was limited within Old World monkeys, including human. These data indicated that the *BLM *partner emerged from an existing promoter in a genomic region that has been stable since the time that marsupials split with the ancestor of modern humans yet has only recently become transcriptionally active in the opposite direction (possibly in the last 5–10 million years).

**Figure 5:**
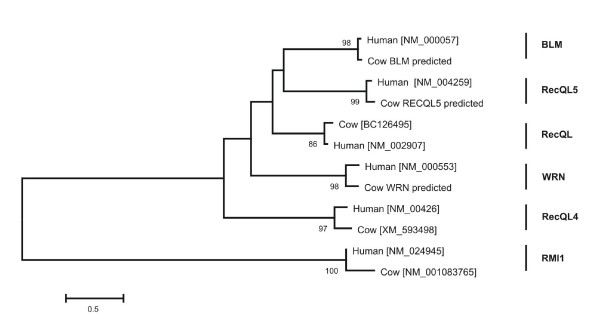
**Phylogenetic tree of 5 RecQ homologous proteins in Human and Cow. A neighbor-joining tree, based on the Dayhoff distance, and rooted with RMI1 proteins**. Rooted with RMI1. Genbank accession numbers are given in square brackets. Putative protein sequences of cow BLM, WRN and RecQL5 are designated as 'predicted' (from the Bovine Genome Database; [8]).

**Figure 6:**
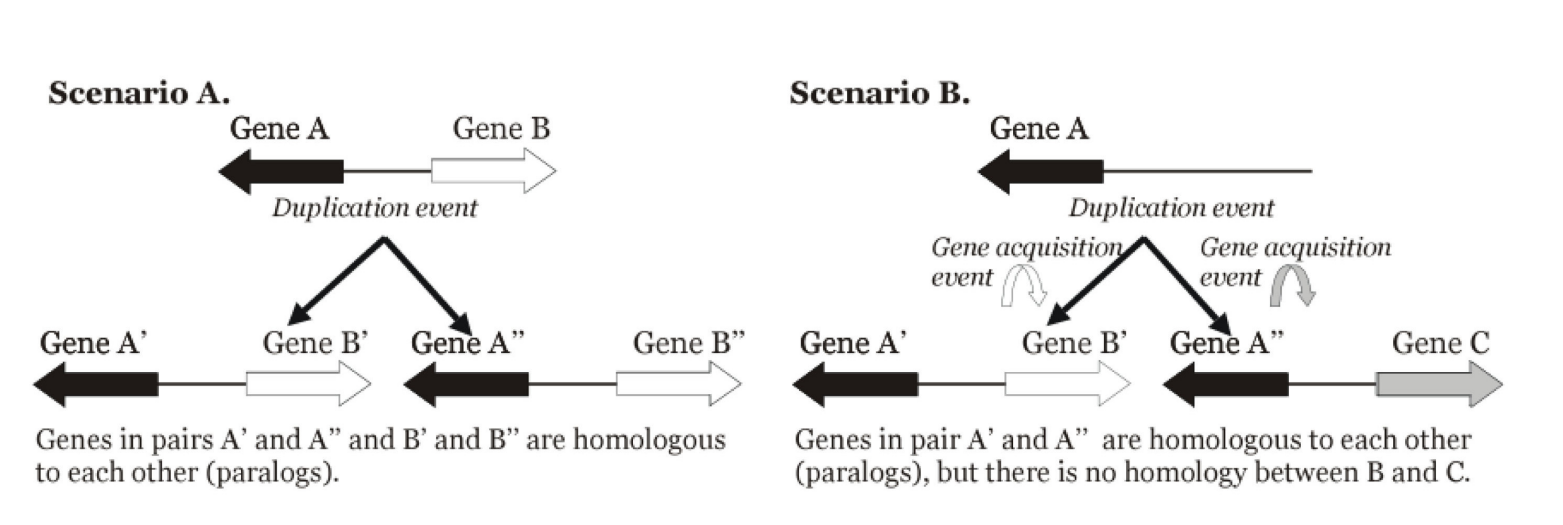
**Possible scenarios for the creation of bidirectional promoters**. Scenario A shows original gene pair being duplicated into a series of paralogous gene-pairs, where respective homology is maintained for all genes, while scenario B shows independent acquisition of gene partners via either a recombination event or *de novo *transcription.

**Figure 7:**
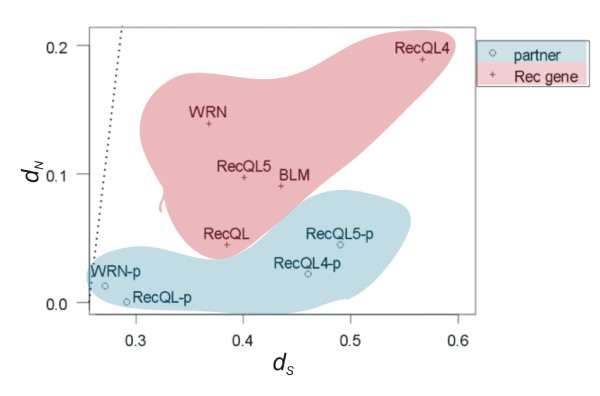
**Relative strength of selective pressure on RecQ helicases and their partners**. The number of nonsynonymous (amino acid altering) nucleotide substitutions per nonsynonymous sites (*d*_*N*_) plotted against the number of synonymous nucleotide substitutions per synonymous sites (*d*_*S*_) are plotted for *RecQ *genes and their partners. The dashed line indicates the neutral expectations (i.e., *d*_*N *_= *d*_*S*_); values below the dashed line indicate purifying selection is in operation, as reflected by *d*_*S *_> *d*_*N*_.

In addition to *BLM*, four other RecQ paralogs exist in the human genome and all were found to have bidirectional promoters. A phylogenetic tree of the five RecQ family members supported their early duplication from an ancestral gene early in metazoan evolution [14] without any gene conversion events that would complicate the evolutionary assessment (Fig. 5). We questioned whether bidirectional promoters had emerged at these sites in a parallel manner, or if the original gene pair duplicated into a series of paralogous gene-pairs that carried along their promoters as part of the duplication event (Fig. 6). To investigate the relationships of the RecQ gene homologs and their partners, the evolutionary distances between human and cow orthologs were computed as *d*_*S *_and *d*_*N *_values (the numbers of synonymous and nonsynonymous substitutions per synonymous and nonsynonymous sites, respectively). For each RecQ gene the rate of synonymous substitutions exceeded that of nonsynonymous substitutions, confirming that purifying selection maintained their respective amino acid sequences (Fig. 7 and Table 3). Divergence at the amino acid level (estimated as Dayhoff distance) was equivalent to that of most protein coding genes in the human genome.

**Table 3:** Pairwise sequence distances between Human and Cow orthologs

	Protein divergence			Nucleotide divergence				
	Dayhoff distance	SE		d_S_	SE		d_N_	SE
					
*BLM*	0.18485	0.01325		0.43527	0.02798		0.09082	0.00591
					
*WRN*	0.29079	0.01614		0.36762	0.02219		0.13946	0.00886
					
*RecQL*	0.09641	0.01175		0.38489	0.04818		0.04467	0.00519
					
*RecQL5*	0.21477	0.0169		0.40091	0.0286		0.09723	0.00571
					
*RecQL4*	0.39271	0.02182		0.56717	0.04907		0.18944	0.01345
					
*RMI1*	0.18976	0.01741		0.27871	0.0302		0.09374	0.01009

(shown are distance values together with standard errors (SE) estimated by the bootstrap method in MEGA4 [22])

In contrast to the relatively high similarity among paralogous RecQ family members, the partners of those genes showed no similarity with one another within the same genomes. Notably, the rate of synonymous substitutions substantially exceeded the rate of nonsynonymous substitutions for all of these orthologous gene pairs (in human-cow comparisons), indicating that purifying selection also maintained these partner sequences (Fig. 7 and Table 4). These results precluded a role for positive selection (i.e., when a duplication was followed by accelerated sequence divergence) as the explanation for all 5 paralogs having bidirectional promoters with unrelated partner genes. Thus, we concluded that each of these bidirectional promoters developed independently, with the *BLM *promoter being the youngest example. Subsequent and ongoing analyses are aimed at identifying the components that could have caused this event. Thus far, no significant sequence alignments have been found between the promoters of the RecQ genes using *blastz *alignments.

**Table 4:** The extent of pairwise nucleotide and amino acid sequence divergence between Human and Cow orthologs of *RecQ *partner genes

Partner of	Protein divergence			Nucleotide divergence			
			
	Dayhoff distance	SE		d_S_	SE	d_N_	SE
			
*BLM*	no significant sequence similarity			no significant sequence similarity			
			
*WRN *	0.02582	0.0098		0.27122	0.03664	0.0127	0.00576
			
*RecQL*	0	0		0.2911	0.07181	0	0
			
*RecQL5*	0.06735	0.016		0.49054	0.05824	0.04479	0.00927
			
*RecQL4*	0.04534	0.0108		0.46026	0.03189	0.02198	0.00406

(shown are distance values together with standard errors (SE) estimated by the bootstrap method in MEGA4 [22])

### Comparative Vertebrate Analyses

**Figure 8:**
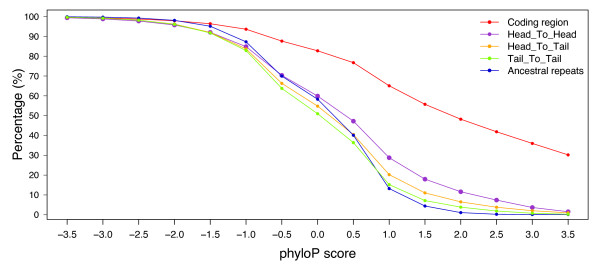
**Sequence conservation in bidirectional promoters**. The 44-way vertebrate phyloP scores are calculated in intergenic regions collected from head-to-head, head-to-tail, or tail-to-tail gene arrangements. Coding regions and ancestral repeats serve as control sets for comparison. These data are plotted as the percentage of bases having phyloP scores equal to or larger than the phyloP score specified on the horizontal axis. Negative scores indicate accelerated change whereas positive scores indicate sequence conservation.

To further address the level of conservation expected at these promoter regions, we examined PhyloP scores [15], which provide a measure of sequence constraint or accelerated change compared to a neutral background model. Multi-species alignments generated from 44 vertebrates were the source for the PhyloP scores (available at the UCSC Human Genome Browser). Coding sequences, which carry a strong signature of sequence conservation, had the highest scores of any dataset we examined. Bidirectional promoters (head-to-head) scored only slightly higher than promoters in head-to-tail arrangements (Fig. 8). The level of conservation decreased further for tail-to-tail genes and ancestral repeats. Thus, bidirectional promoters are not maintained as highly conserved sequences in mammalian genomes, despite their close positioning between genes that are under negative (purifying) selection. Instead, these promoters tolerate sequence divergence, which is consistent with observations of promoters in general [5]. We found that a core set of regulatory factors is significantly enriched in the orthologous promoter set compared to the tail-to-tail regions (Additional file 2).

**Figure 9:**
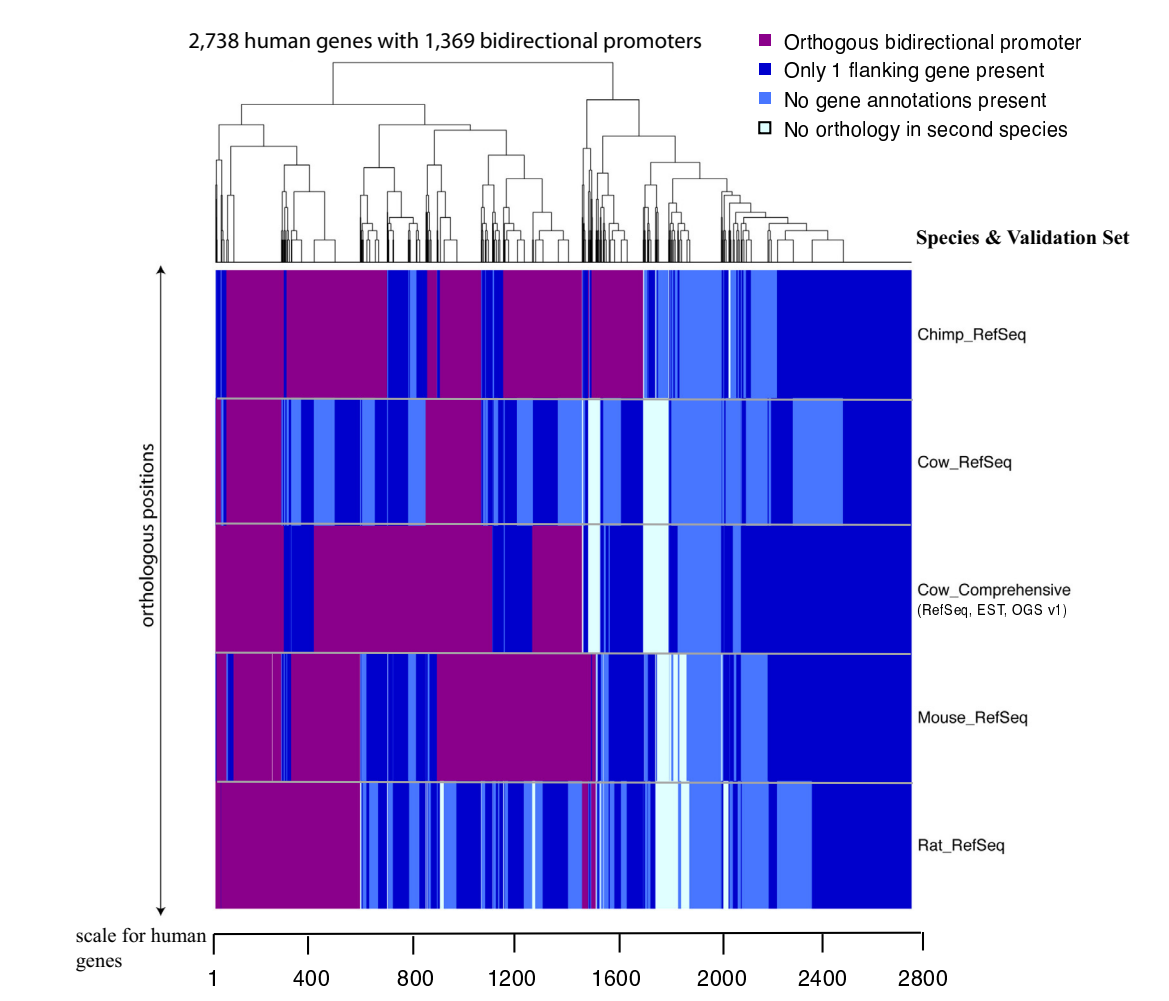
**Comparative mapping of bidirectional promoters across species at orthologous positions**. The reference set of bidirectional promoters is from 2,738 individual human protein coding genes (regulated by 1,369 bidirectional promoters). These regions were mapped to the species shown on the right of the panel. The orthology assignments were validated using ResSeq annotations in the non-human species, except for Cow_Comprehensive, which contained RefSeq, EST and OGS v1 annotations from cow. The heatmap is clustered keeping the vertical columns intact across all datasets to retain the relevance to the reference position in human. The order of the reference human gene set is dependent on the outcome of the clustering algorithm. The scale of the heat map is depicted as the number of human gene pairs used for predictions. Vertical bars representing each bidirectional promoter are colored purple when validated as orthologous in other species, royal blue when only one of the two flanking genes is annotated in cow, light blue when neither gene is annotated in cow, or off-white when no orthology is present for the human region.

Our approach of mapping orthologous bidirectional promoters across species was expanded to produce a 5-species map of validated bidirectional promoters (Fig. 9). The dataset containing 1,369 bidirectional promoters of human protein-coding genes was mapped to the other genomes and validated using RefSeq annotations from each of those genomes, keeping the 1,000 bp maximal intergenic distance requirement. Nearly all regions showed orthology at one or both of the genes, except 10% of regions in chimp, 6% in cow, 5% in mouse and 8% in rat, respectively. These locations represent complete changes in genomic content between species. The remaining regions contained orthology but validated at relatively low rates as illustrated by the magenta areas in the heat map. Nevertheless, these promoters were validated across species in numbers consistent with the gene annotations available for each genome: 934 promoters in chimp, 828 in mouse and 494 in rat, respectively (using RefSeq annotations only). These data indicate that many 5' UTRs remain to be annotated in mammalian genomes, moreover, some protein coding genes are absent from these gene collections.

## Discussion

Bidirectional promoter analyses contribute many benefits to genomic annotations including predictions of unannotated genes and 5' UTRs in mammalian genomes. No other methods exist to predict UTRs across species, and conventional techniques to align ESTs from other species perform poorly in the divergent UTR sequences. In addition to 5' UTR annotations, the conserved architecture of bidirectional promoters enables annotation of orthologous promoters as well as identification of missing coding annotations in other mammals. Our previous work reported that similar intergenic distances were present at orthologous positions of bidirectional promoters in pairwise comparisons between human and mouse or human and chimp. Thus, the finding that the human and cow comparison showed fewer bidirectional promoters in the bovine genome was attributed to the early stage of the bovine annotation effort, in which 5' UTRs of genes have not been fully characterized. As the depth and coverage of transcribed regions in the cow genome increase, both the in-species annotations and cross-species validations of bidirectional promoters will benefit. Consistent with this idea, our analyses strongly advocate continued efforts towards defining the 5' ends of genes, in order to expedite the annotation of adjacent regions, which contain promoter sequences. The resulting information will enable downstream analyses of conservation of regulatory networks that act through the same transcription factor binding sites. Despite the limitations imposed by the shallow depth of transcripts available in the bovine genome, our data provide strong evidence that bidirectional promoters can be mapped across species using conservation of gene synteny to rapidly annotate these functional regions in non-human genomes. Furthermore, the benefit of mapping these promoters independently in other species is that new bidirectional promoters can be reciprocally predicted and validated in the human genome. We were able to use bidirectional promoters to identify chromosomal rearrangements, which harbor species-specific transcripts with robust evidence and biological intrigue. For example, RecQ genes originated via duplication events that created 5 paralogs early in the evolution of metazoans, but their bidirectional partners are unrelated to each other. Therefore, the emergence of bidirectional promoters in this gene family was not a passive result of duplication of the original gene pair bringing the promoter along with it. This conclusion is apparent from the lack of similarity among the partner genes. Moreover, *d*_*N*_/*d*_*S *_analyses show that the partners are under strong purifying selection and have not undergone accelerated change, which would have masked their original similarities if they had been paralogs. Any tendency towards rearrangement by these genes is no longer apparent, as each gene partner has remained stably associated with its RecQ paralog for more than 80 million years. Retrotransposition could be another mechanism bringing RecQ genes near their partners or *vice versa*, but does not explain the *BLM *gene pairing. The partner of the *BLM *gene is unique to Catarrhini and has only recently evolved, however, the underlying genomic sequence is orthologous to opossum sequence, precluding a recent introduction of the partner gene into the region. Given these data, we hypothesize that elements in or near the *RecQ *promoters are responsible for initiating transcription in the opposing direction. Recent work by Core et al. 2008 [16] and Seila et al. 2008 [17] demonstrates that promoters can load RNA Pol II in both the forward and reverse directions to maintain a short region of open chromatin. We propose that in some cases, this phenomenon could provide a mechanism for generating novel, full-length transcripts that are spliced and polyadenylated in a species-specific manner.

## Conclusion

We have produced a record of orthologous bidirectional promoters in 5 placental mammals (human, chimpanzee, bovine, murine, and rat). Furthermore, we addressed the evolution of new genes that can be identified by mapping bidirectional promoters across species. Continued work on the development of a cross-species regulatory map for these promoters is likely to reveal additional information about transcripts that are not only unique to individual species, but also functionally relevant.

## Methods

The bovine gene annotations from OGS v1 are available at . All other annotations were obtained from the UCSC Human Genome Browser. PhlyoP scores were also obtained from the Human UCSC Genome Browser.

A multi-stage approach to mapping orthology at bidirectional promoters was developed. For example, orthology assignments are strongest in coding regions. Therefore, we began by mapping single human genes regulated by bidirectional promoters from the Known Genes annotations [18] of the UCSC human genome assembly hg18. Orthology assignments were determined using *multiz *alignment information [19], the "chains and nets" data from the UCSC Human Genome Browser mysql tables [20]. Chains in the Genome Browser represent sequences of gapless aligned blocks. Nets provide a hierarchical ordering of those chains. Level 1 chains, which contain the longest, best-scoring sequence chains that span any selected region, were the only ones considered in this analysis. Given a human gene, our approach examined whether it fell within an orthologous region defined by level 1 alignment data without knowledge of the exact position within an alignment or relative to a gap. In a subsequent step, we intersected the positions of gaps and exons of each gene to ensure that the exons fell into alignable positions across species.

After determining the orthology assignments using the UCSC chains and nets data, we used the RefSeq, spliced ESTs, or OGS v1 annotations from cow to validate predictions from the human dataset. RefSeq genes represent mostly protein-coding genes and therefore were verified by chains and nets alignments, followed by confirmation of protein identity in both species. Spliced ESTs carried less descriptive information than protein coding genes and therefore were validated in the second species by their presence in an orthologous region, showing conserved synteny of the two genes within that gene-pair, and meeting the criteria of less than 1,000 bp of intergenic distance between those transcripts. Our method for mapping bidirectional promoters in spliced EST datasets is described in more detail in a previous publication [4]. If the program verified evidence for orthology and conserved-syntenic gene arrangement, then the orthologous bidirectional promoter was confirmed. After orthologous assignments were confirmed for pairs of human genes, the reciprocal assignments were analyzed from cow to human, using a similar process.

Heat maps were generated to represent the orthologous positions of bidirectional promoters. The scale of the map is designated by the number of human genes that were evaluated in a linear distance on the map, due to the fine gradation of the illustrated data. Circumstances in which orthologous bidirectional promoters were not identified included: (A) the presence of one flanking gene but not both, (B) no annotations for either gene in the orthologous genomic region, or (C) no orthologous genomic region was identified. Additional scenarios were possible, but were not presented here. Bidirectional promoters that were validated at the same orthologous position across multiple species are presented as columns of purple color. The heatmap is clustered by similar color groups, while maintaining the column of information at each position.

The phylogenetic tree of protein sequences showing 5 members of the RecQ gene family from human and cow was reconstructed using the neighbor-joining method [21] based on the Dayhoff distance implemented in the MEGA4 program [22]. The RMI1 sequences from human and cow were used as an outgroup to root the tree. The reliability of the internal branches was evaluated using 1,000 bootstrap replications [23]. The number of synonymous (*d*_*S*_) and nonsynonymous (*d*_*N*_) substitutions per synonymous and nonsynonymous site, respectively, was computed using Nei-Gojobori method [24].

## Authors' contributions

MQY designed and implemented the software to map bidirectional promoters from gene annotations, mapped sequences across species, and helped write the manuscript. HP carried out the evolutionary analyses of RecQ helicase genes and helped write the manuscript. LE conceived of the analysis and helped write the manuscript, DL and HL contributed expertise on the cow-specific rearrangement, JR annotated the annotations for 10 cow genes using the Otterlace annotation system.

## Supplementary Material

Additional File 1 **Supplemental figure S1**. Validation of human bidirectional promoters in cow. Click here for file

Additional File 2 **Supplemental table S1**. Conserved motifs enriched in head-to-head regions compared to tail-to-tail regions. Click here for file
