# Supplementary material for: Cross-species mapping of bidirectional promoters enables prediction of unannotated 5' UTRs and identification of species-specific transcripts
Source: BMC Genomics. 2009 Apr 24;10:189. doi: 10.1186/1471-2164-10-189 (PMC2688522; doi:10.1186/1471-2164-10-189)
Supplement: Additional File 1 — Supplemental figure S1. Validation of human bidirectional promoters in cow. [file 1471-2164-10-189-S1.pdf]

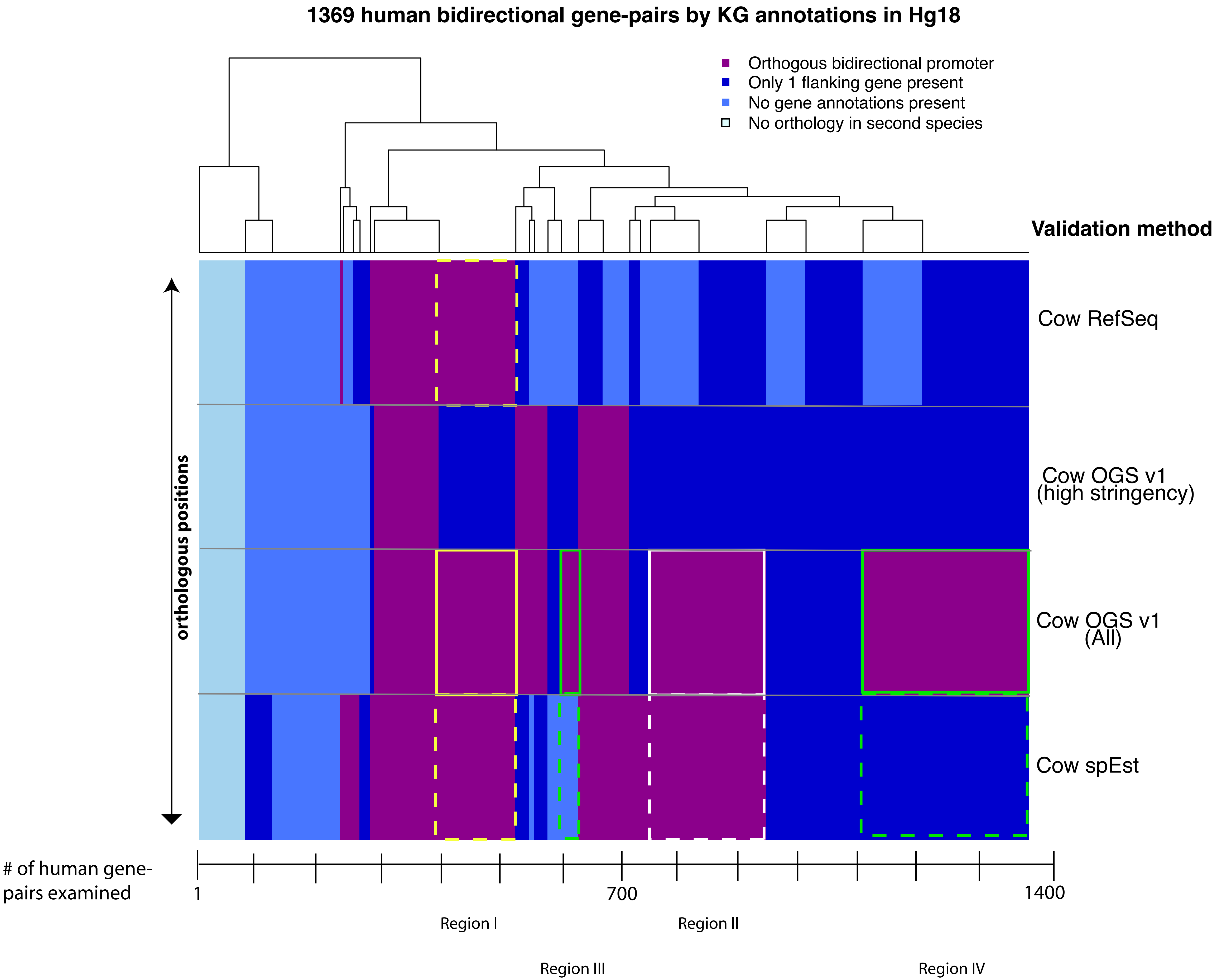
1369 human bidirectional gene-pairs by KG annotations in Hg18
Orthogous bidirectional promoter
Only 1 flanking gene present
No gene annotations present
No orthology in second species
Validation method
Cow RefSeq
Cow OGS v1 (high stringency)
Cow OGS v1 (All)
Cow spEst
orthologous positions
# of human gene-pairs examined
1
700
1400
Region I
Region II
Region III
Region IV

Figure 4

Supplemental Figure 1. Validation of human bidirectional promoters in cow. Bidirectional promoters from 1,369 annotated human protein coding gene pairs were mapped to the cow genome. The data were validated using bidirectional promoters identified from cow datasets listed on the vertical axis of the chart. The heatmap is clustered keeping the vertical columns intact across all 4 datasets to retain the relevance to the reference position in human. The order of the reference human gene set is dependent on the outcome of the clustering algorithm. The scale of the heat map is depicted as the number of human gene pairs used for predictions. Vertical bars representing each bidirectional promoter are colored purple when validated as orthologous in cow, royal blue when only one of the two flanking genes is annotated in cow, light blue when neither gene is annotated in cow, or nearly white when no orthology is present for the human region. Regions indicating the presence of 5' UTR annotations were determined by comparison to cow coding sequence annotations from the OGS v1 dataset. All datasets contained high stringency bidirectional promoter annotations except OGS v1 (ALL), which included low stringency predictions. Low stringency bidirectional promoters in OGS v1 were validated as high stringency regions by comparison to high stringency ResSeq and spliced EST data (Region I) or EST data only (Region II), as indicated by the boxed regions. Many low stringency validations currently lack EST evidence in the cow (Regions III and IV; green dashed boxes).
