# Supplementary material for: Cross-species mapping of bidirectional promoters enables prediction of unannotated 5' UTRs and identification of species-specific transcripts
Source: BMC Genomics. 2009 Apr 24;10:189. doi: 10.1186/1471-2164-10-189 (PMC2688522; doi:10.1186/1471-2164-10-189)
Supplement: Additional File 2 — Supplemental table S1. Conserved motifs enriched in head-to-head regions compared to tail-to-tail regions. [file 1471-2164-10-189-S2.doc]

Supplemental Table 1. Conserved motifs enriched in head-to-head regions compared to tail-to-tail regions.

| **Conserved_in_All(H)** | **in_Human(H)** | **Score(H)** | **Motif** | **Conserved_in_All(T)** | **in_Human(T)** | **Score(T)** |
| --- | --- | --- | --- | --- | --- | --- |
| 37 | 68 | 0.54 | V$ELK1_02 | 0 | 10 | 0 |
| 31 | 65 | 0.48 | V$NRF2_01 | 0 | 9 | 0 |
| 39 | 89 | 0.44 | V$SP1_Q6 | 3 | 27 | 0.11 |
| 20 | 50 | 0.4 | V$NFY_Q6 | 0 | 15 | 0 |
| 48 | 94 | 0.51 | V$CETS1P54_01 | 9 | 32 | 0.28 |
| 44 | 88 | 0.5 | V$CETS1P54_02 | 9 | 34 | 0.26 |
| 47 | 98 | 0.48 | V$SP1_01 | 11 | 41 | 0.27 |
| 24 | 51 | 0.47 | V$CAAT_01 | 5 | 25 | 0.2 |
| 39 | 85 | 0.46 | V$AP2_Q6 | 0 | 22 | 0 |
| 40 | 89 | 0.45 | V$GC_01 | 5 | 27 | 0.19 |
|  |  |  |  |  |  |  |

Conserved_In_All (H): The number of head_to_head regions containing the motif in orthologous regions among 5 mammals;

In_Human (H):  The total number of human head_to_head regions having the motif.

Score (H) = Conserved_In_All (H) / In Human(H).

The labels (Conserved_In_All (T), In_Human (T),Score (T)) have the same meaning for tail_to_tail regions.
